# Supplementary material for: CNeuroMod-THINGS, a densely-sampled fMRI dataset for visual neuroscience
Source: Sci Data. 2026 Jan 29;13:141. doi: 10.1038/s41597-026-06591-y (PMC12858831; doi:10.1038/s41597-026-06591-y)
Supplement: Supplementary file 1 — Supplementary Information [file 41597_2026_6591_MOESM1_ESM.pdf]

CNeuroMod-THINGS, a densely-sampled fMRI dataset for visual neuroscience

Supplementary Information

S1. Misordered sessions ………………………………………………………………….. p.2

S2. Manual Image annotation ……………………………………………………………….. p.4

S1. Misordered sessions

Due to errors at the console, a small number of sessions were administered out of their pre-planned order, which introduced irregular patterns of repetition (in delays and rep numbers) for a subset of images. Data from affected sessions may include unexpected memory interactions; as such, we recommend that they be omitted from memory-specific analyses. Irregular sessions are flagged to facilitate this decision. The "atypical", "atypical_log" and "not_for_memory" columns in run *events.tsv files can be used to filter out trials (*events.tsv files are found under cneuromod-things/THINGS/fmriprep/sourcedata/things under subjects-specific session directories; file columns are defined in this document: https://github.com/courtois-neuromod/things/blob/d794c31a88bb8b827b023185b127a6f95c5d6214/task-things_events.json)

Table S1: session-level deviations from pre-planned order of presentation

	**sub-01, ses-17**
	**Issue**: Planned runs were administered in the following order: 1, 2, 3, 5, 6, 4 (as labeled in the dataset). The session also included a “false-start” for which a run was interrupted and re-done in full, introducing additional repeats for a small portion of the session.<br><br>**Fix**: The condition (“seen”/”unseen”) and sub-condition (e.g., “seen-between”, ”seen-between-within”) columns and the subject’s performance metrics (“error”) are corrected in the *events.tsv files to reflect the order in which the runs were administered (rather than planned). Condition labels and performance metrics were also corrected where appropriate for the few trials affected by the false-start (e.g., no error was counted if the subject recognized a novel image accidentally shown during the false-start).<br><br>**Impact:** Minimal impact on the overall structure of the experiment, and no impact on previous or subsequent sessions.
	**sub-03, ses-14**
	**Issue:** Stimuli prepared for sub-02, ses-04 were accidentally shown instead of the stimuli planned for sub-03, ses-14.<br><br>**Fix:** BOLD and behavioural data from the accidental completion of sub-2’s ses-04 by sub-03 are excluded from the dataset. Sub-03’s planned ses-14 was correctly re-run two weeks later with the proper stimuli, and those data are included in the dataset.<br><br>**Impact:** This mistake increased the delay between sub-03’s ses-13 and ses-14 and potentially interfered with the memory task by presenting additional stimuli between sessions meant to be consecutive. However, because the extra session was made of stimuli already presented to sub-03 earlier in the study, it did not alter planned repetition patterns for any of the previous or subsequent sessions.
	**sub-03, ses-22 to ses-25**
	**Issue:** Sub-03’s planned ses-25 was accidentally run instead of sub-03 ses-22.

	**Fix:** After this mistake, ses-22 (with the correct stimuli), ses-23, ses-24, and then ses-25 (repeated a second time in its proper order) were administered with their planned stimuli to correct course. Data from the first ses-25 (administered out-of-order) are dropped from the dataset. Condition labels and performance metrics were corrected for ses-24, ses-25 and ses-26 to reflect the altered repetition pattern accurately (e.g., higher numbers of repetitions and atypical sub-condition labels like “seen-within-between-within-between” were introduced for some images).<br><br>**Impact:** This mistake introduced delays and interference between sub-0-3’s ses-21 and ses-22, and atypical patterns of repetitions for ses-24, ses-25 and ses-26.
	**sub-06, ses-20**
	**Issue:** Stimuli from sub-01’s ses-20 were accidentally shown for sub-06’s session 20.<br><br>**Fix:** After this mistake, sub-06 ses-21, ses-22 and so-on were acquired with their planned stimuli. Data from ses-20 administered with the wrong (sub-01’s) stimuli are included in the dataset (no repeat was made of ses-20 with the planned stimuli). By chance, the stimuli accidentally shown in sub-06 ses-20 were planned for that 6-session block so that deviations from the task were contained to that block. Condition labels and performance metrics were corrected for ses-20 to ses-26 (e.g., introducing higher numbers of repetitions and atypical sub-condition labels). All sessions (20-26) are included in the dataset with their corrected labels and performance metrics.<br><br>**Impact:** This mistake introduced atypical patterns of repetitions for ses-20 to ses-26.
	**sub-06, ses-19**
	**Issue:** Due to scanner issues, sub-06 ses-19 included two “false-starts” for which a run was interrupted and re-done in full, introducing additional repeats for a small portion of the session.<br><br>**Fix:** Corrections were made in the *events.tsv files to the condition and performance metrics for the few trials affected by these additional repeats.<br><br>**Impact:** This issue introduced atypical repetition patterns (additional repeats) for the task segments that were shown twice.

S2. Manual Image annotation

Table S2 defines the boolean flags introduced by author MSL to annotate the content of the stimulus set.

Table S2. Manual image annotation flags

Flag name	Description
face	Contains any face, whole or partial (e.g., eyes or smile), central or in the periphery (incidental to the image's main focus). Faces can be human or not, real or artificial (a doll's face, a cartoon cat face, a face reflected in a soap bubble).
body	Contains any body (which may include a face) or non-face body part(s), central or in the periphery. Bodies can be human or not, real or artificial (a robot's body, an octopus tentacle, an artificial limb), clothed or not (e.g., a person's legs in an image featuring pants, a gloved hand holding a wine glass, a head with visible shoulders and torso).
human face	Contains any face or portion of a face with human features, central or in the periphery, real or artificial (a doll's face, a cartoon of a person's face, etc).
human body	Contains any human body or non-face human body part, central or in the periphery, real or artificial (a drawn hand).
non-human mammal face	Contains any face with non-human mammalian features, central or in the periphery, real or artificial (a cartoon dog face).<br>Includes: faces of rodents, dogs, felines, cows, deers, etc.<br>Excludes: faces of insects, reptiles, fish, birds, sea mammals* (e.g., dolphins and whales).
non-human mammal body	Contains any body or non-face body part from a non-human mammal, central or in the periphery, real or artificial (a robot dog leg).<br>Includes: rodents, felines, cows, deers, etc.<br>Excludes: insects, reptiles, fish, birds, sea mammals* (e.g., dolphins and whales).
central face	Contains a face or a portion of a face (human or not, real or artificial) within the image's central focus. E.g., An image featuring a person jumping on a pogo stick is considered central if the face is visible, while faces of random spectators in the background are not. Although the face does not need to be in the middle of the image to be "central" per se, the face should be visible when gazing at a central fixation cross, and it must be part of the image's main focus of interest.
central body	Contains a body or non-face body part within the image's central focus. E.g., an image featuring a hand holding an item of interest is central, while a silhouette visible next to an aircraft carrier seen from afar is not. Although the body (part) does not need to be in the center of the image to be "central", it should be visible when gazing at a central fixation cross, and it must be part of the image's main focus of interest.

artificial face	Contains any representation of a human, animal or humanoid face that is not a real (living) face. Eg., a doll face or mannequin head, a cartoon bird face, a robot with facial features like eyes and a smile, an action figure, a painting or a photo of a face on a banner.
artificial body	Contains any representation of a human, animal or humanoid body or body part that is not a real (living) body (part). Eg., a statue, a prosthetic leg, a robotic hand, a drawing of a bird on a teapot.
scene	An item pictured in an environment with a background and a foreground that gives the sense of a place around it. A scene includes scenery, a view point and some perspective. It can be the image of a large object in a specific setting, like an aircraft in a hangard, a sofa in the middle of a living room, an anchor by a waterfront, an elephant at the zoo or a person skateboarding in a busy park.
rich background	An image of an object taken from closer than a scene, but that still includes items, people or animals clearly visible in the background. E.g., a backpack on someone's back walking away toward some trees, a tool in a garage with equipment visible behind it, a plant in a garden surrounded by other plants, a beer glass held by a person sitting between others, an apple on a table with orchard trees behind it.
lone object	The featured object is shown centrally with no additional objects visible in the periphery or background. Not only is the object shown by itself, but the empty background is uniform and minimally textured (no carpet, dinner mat or wooden fence behind) or blurred so that only the lone object is in focus. Note that objects can either be shown with zero background (the "lone objects" flag), with minimal background (e.g., an apple in a basket on a table), with noticeable background (the "rich background" flag) or within a scene (with background and perspective).

*Although they are mammals, sea mammal's features are very different from those of humans, hence this categorical choice.
